# Epithelial Uptake of Flagella Initiates Proinflammatory Signaling

**DOI:** 10.1371/journal.pone.0059932

**Published:** 2013-03-20

**Authors:** Dane Parker, Alice Prince

**Affiliations:** Department of Pediatrics, Columbia University, New York, New York, United States of America; Cincinnati Children’s Hospital Medical Center, United States of America

## Abstract

The airway epithelium serves multiple roles in the defense of the lung. Not only does it act as a physical barrier, it acts as a distal extension of the innate immune system. We investigated the role of the airway epithelium in the interaction with flagella, an important virulence factor of the pathogen *Pseudomonas aeruginosa*, a cause of ventilator associated pneumonia and significant morbidity and mortality in patients with cystic fibrosis. Flagella were required for transmigration across polarized airway epithelial cells and this was a direct consequence of motility, and not a signaling effect. Purified flagella did not alter the barrier properties of the epithelium but were observed to be rapidly endocytosed inside epithelial cells. Neither flagella nor intact *P. aeruginosa* stimulated epithelial inflammasome signaling. Flagella-dependent signaling required dynamin-based uptake as well as TLR5 and primarily led to the induction of proinflammatory (*Tnf*, *Il6*) as well as neutrophil (*Cxcl1*, *Cxcl2*, *Ccl3*) and macrophage (*Ccl20*) chemokines. Although flagella are important in invasion across the epithelial barrier their shedding in the airway lumen results in epithelial uptake and signaling that has a major role in the initial recruitment of immune cells in the lung.

## Introduction

*Pseudomonas aeruginosa* is a major opportunistic pathogen associated with infection in compromised hosts. It is one of the major causes of ventilator associated pneumonia (VAP), a common and costly complication of modern intensive care [1]. Following colonization of the upper respiratory tract and often biofilm formation, these opportunistic pathogens are aspirated into the lower airways and initiate pneumonia. Some of these infections are complicated by bacteremia and sepsis, especially those attributed to organisms expressing specific type III secreted toxins [2]. The type III secreted toxins and motility are conserved attributes associated with the pathogenesis of acute *P. aeruginosa* pneumonia in this setting.

Motility and attachment are major functions attributed to *P. aeruginosa* flagella thought to be relevant to the pathogenesis of pneumonia. *fliC* mutants have been shown to cause less mortality than wild type organisms in murine models of acute pulmonary infection as they fail to disseminate and are associated with more focal infection in the lung [3], [4]. Flagella are ligands for epithelial cells in model systems, interacting, at least in vitro, with baso-lateral receptors-heparan sulfate proteoglycans [5]. Phagocytic cells are able to recognize motility as strains expressing non-functional flagella are able to evade phagocytosis [6], [7], [8].

Perhaps the best characterized receptors for flagella are those involved in their potent immunostimulatory activities [9]. As highly conserved PAMPs there are at least two discrete signaling systems dedicated to flagellin recognition, TLR5 [10], [11] and the NLRC4/IPAF inflammasome [12], [13], [14], [15]. TLR5 can be present apically in the airway epithelium [16], [17], in contrast to the gut in which TLR5 is exclusively found on the basolateral side [18], which is linked to the activation of NF-κB and the induction of proinflammatory chemokines such as IL-8 and cytokines [10], [11], [16]. Recent structural studies suggest that the flagellin epitope that interacts with TLR5 is not exposed in intact flagella [19] as it is involved in FliC oligomerization [20]. Thus, flagellin monomers are required for TLR5 recognition, implying a requirement for some type of proteolytic processing. In models of airway infection, *Tlr5* ^−/−^ mice have very modest defects in early immunostimulation and no significant defects in *P. aeruginosa* clearance or survival [11], [21]. Only in the absence of TLR4 and TLR5 do mice become susceptible to *P. aeruginosa* infection [11], [22].

The mechanisms of activation of the NLRC4/IPAF inflammasome by flagellin have been extensively characterized [12], [13], [14], [15], [23]. Flagellin, as well as the PscI component of the type three secretion system (TTSS), directly interacts with the NLRC4 inflammasome, resulting in the production of IL-1ß and IL-18 both potent proinflammatory cytokines that can contribute to pulmonary pathology [23], [24], [25], [26], [27]. The inflammasome components are cytosolic, thus this signaling cascade requires the delivery of flagellin to the cytosol, a process that must accompany the processing of intact bacteria or isolated flagellins by macrophages [28]. Although some components of the inflammasome are detectable in airway epithelial cells [29], there has not been evidence of epithelial inflammasome activation in response to *P. aeruginosa* infection in vitro [30]. The biological rationale for these redundant signaling mechanisms is not fully understood, but may reflect the distinct immunological functions carried out by immune cells, that have a major phagocytic function versus epithelial cells that maintain both a physical and immunological barrier. Activation of the inflammasome, induction of caspase-1 activity and pyroptosis of epithelial cells would likely result in a breach of the epithelial barrier and further contribute to *P.aeruginosa* invasive infection.

In the pathogenesis of airway infection, many opportunistic pathogens such as *P. aeruginosa* are entrapped in mucin and often shed surface components including LPS, pili and flagella. In contrast to other epithelial surfaces, particularly the gastrointestinal epithelium, exactly how flagella are sensed and interact with polarized airway epithelial cells has not been well studied. Given the multiple potential interactions of flagella/flagellins and specific epithelial and immune signaling cascades, we determined the role of flagella in; epithelial transmigration, characterized how flagellins affect the barrier function of airway epithelia: establishing their effects on epithelial tight junctions, their ability to stimulate epithelial inflammasome signaling, as well as the signaling pathways associated with TLR5 stimulation.

## Results

### Flagella Mediate *P. aeruginosa* Transmigration Across the Airway Epithelial Barrier

**Figure 1:**
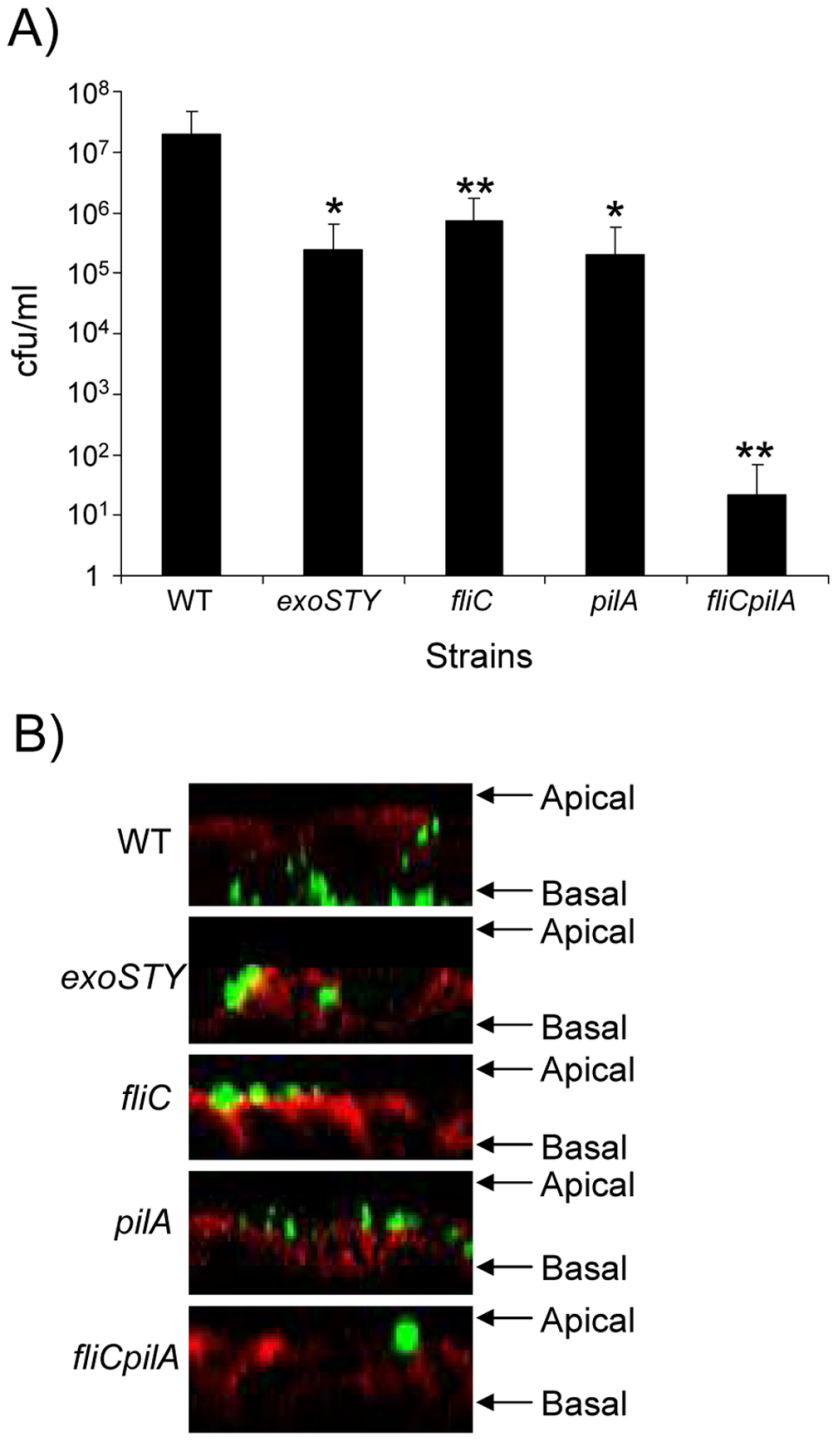
Flagella are required for transmigration across airway epithelial cell barriers. A) Bacteria (1–2×10^7^ cfu) were incubated with 16HBE cells for 4 h before enumerating cfus in the basal compartment. Data are the average of three independent experiments (n = 15). *P<0.05, **P<0.01 students t test compared to WT. B) Z-section from confocal microscopy of GFP expressing *P. aeruginosa* WT and mutant strains migrating across 16HBE cells. E-cadherin is stained red.

In the context of ventilator-associated pneumonia, *P. aeruginosa* colonizing the respiratory tract can initiate invasive infection causing pneumonia, bacteremia and sepsis. Previous studies have demonstrated the importance of the type III secreted toxins (TTST), including the ADP ribosylating activity of *P. aeruginosa* in modifying the epithelial cytoskeleton to facilitate bacterial transmigration across the airway epithelial barrier [31]. We hypothesized that flagella were also involved in bacterial transmigration across the epithelial barrier. Using human 16HBE airway epithelial cells grown in a polarized fashion, we quantified the number of organisms of a 1–2×10^7^ cfu inoculum of strain PAK able to cross an intact epithelium (Fig. 1A). We confirmed the involvement of the TTSS in facilitating transmigration across the epithelium, as almost 2 logs fewer of a ΔSTY mutant were recovered from the basal compartment of the transwells than the wild type control. Pilin dependent bacterial adherence, which is required for TTSS [32] was also involved; as a *pilA* mutant was as impaired as the ΔSTY mutant in transmigration. Lack of FliC similarly inhibited *P. aeruginosa* transmigration across the monolayer (96% reduction, P<0.001). Despite the effects of the type III toxins the *fliCpilA* mutant, unable to attach and inject the TTST or to swim, was the most significantly impaired in epithelial transmigration, almost completely incapable of transmigration (10^6^ reduction, P<0.05).

We confirmed the expected interactions of these mutants and the polarized airway cells by confocal imaging (Fig. 1B). Following a four hour incubation with the monolayers WT PAK (green) was observed primarily at the basal aspects of the monolayer as well as penetrating through the monolayer. The TTST mutant was observed associated with the monolayer, but did not reach the basal surface of the transwells. The *fliC* and *pilA* mutants accumulated at the apical surface; consistent with the function of either flagellin or pilin in attachment, but lacking either motility (*fliC*) or the ability to inject type III toxins (*pilA*) did not penetrate across. The *fliCpilA* was also observed only on the apical side of the monolayer (Fig. 1B).

### Purified Flagella do not Alter the Properties of the Epithelial Junctions

**Figure 2:**
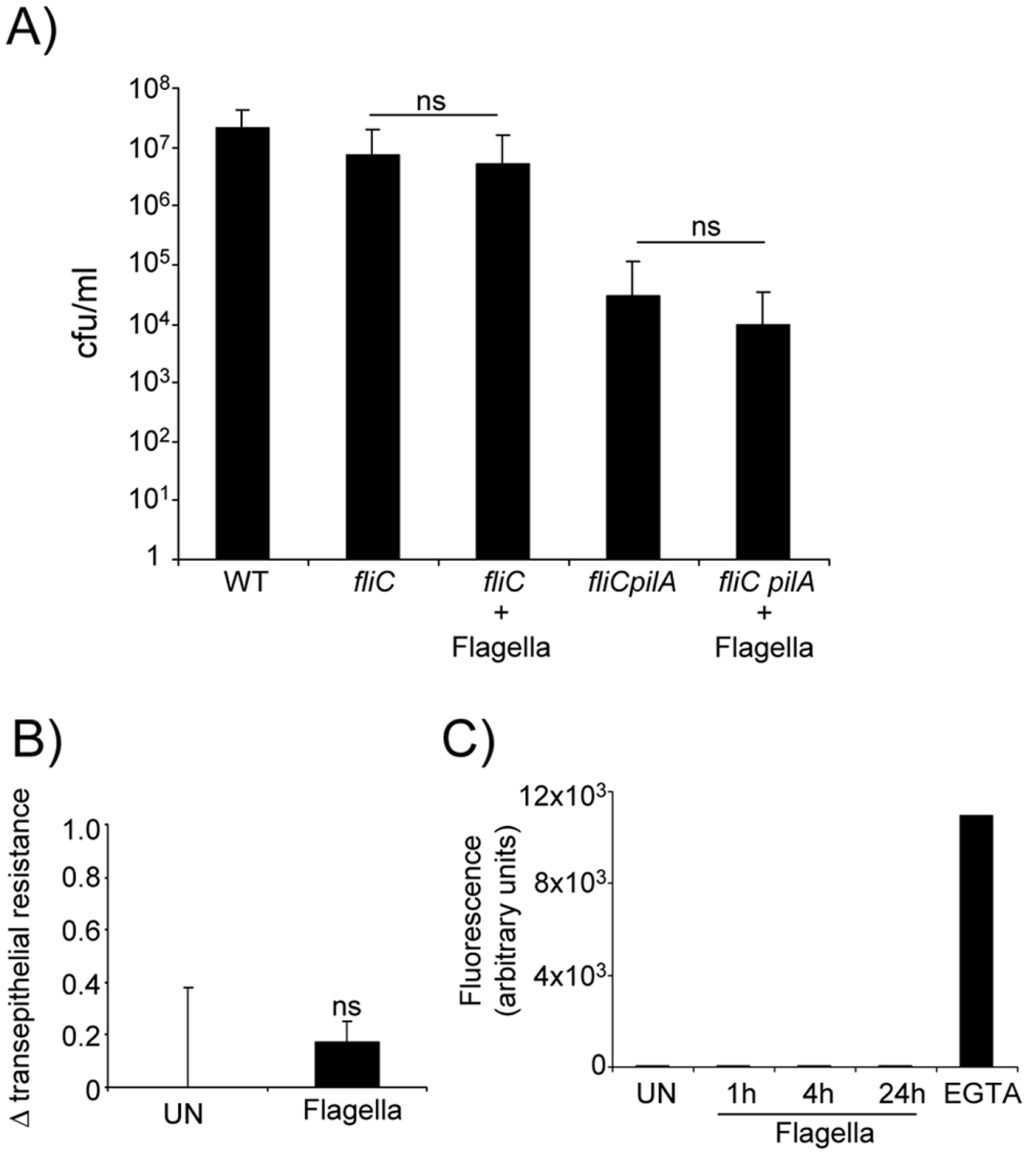
Flagella do not alter the barrier of the airway epithelium. A) Transmigration of *fliC* and *fliCpilA* strains across polarized 16HBE cells was examined with and without the additional of exogenous flagella. n = 12. B) The transepithelial resistance of polarized 16HBE cells was measured after 4 h of flagella stimulation. n = 6. C) The ability of fluorescent dextran to migrate across 16HBE cells to the basolateral compartment was monitored after addition of flagella for varying times. EGTA was used as a positive control. n = 6. ns-not significant.

*P. aeruginosa* movement across an intact epithelial barrier appears to require pilin-mediated attachment, the activity of the type III secreted toxins and the expression of *fliC*. While the motility function associated with *fliC* expression is most likely responsible for bacterial transmigration, given the known signaling functions associated with flagellin, we sought to determine if isolated flagella alter the barrier properties of polarized airway cells. This could occur either as a direct effect or as a consequence of downstream signaling as has been observed for TLR2 stimulation which causes calpain activity and cleavage of junctional proteins [33]. We first explored whether isolated flagella could complement the transmigration defect of the *fliC* mutant (Fig. 2A). Purified flagella were added back to the *fliC* and to the *fliCpilA* mutants and epithelial transmigration quantified. As shown, there was no detectable complementation of the transmigration defect of either mutant. To establish directly if flagella themselves alter the barrier properties of airway epithelial cells, we monitored the translocation of 3, 000 MW dextran (as a marker for barrier function) from the apical to the basolateral compartment of human airway epithelial cells at 1 and 4 hours following the addition of *P. aeruginosa* flagella (Fig. 2B), as well as changes in transepithelial resistance over this period of time (Fig. 2C). While the epithelial barrier maintained an initial resistance of on average 424 ohms/cm^2^, there was no evidence that flagella directly changed the barrier properties of the airway epithelium.

### Endocytosis of Flagella by Airway Epithelial Cells

**Figure 3:**
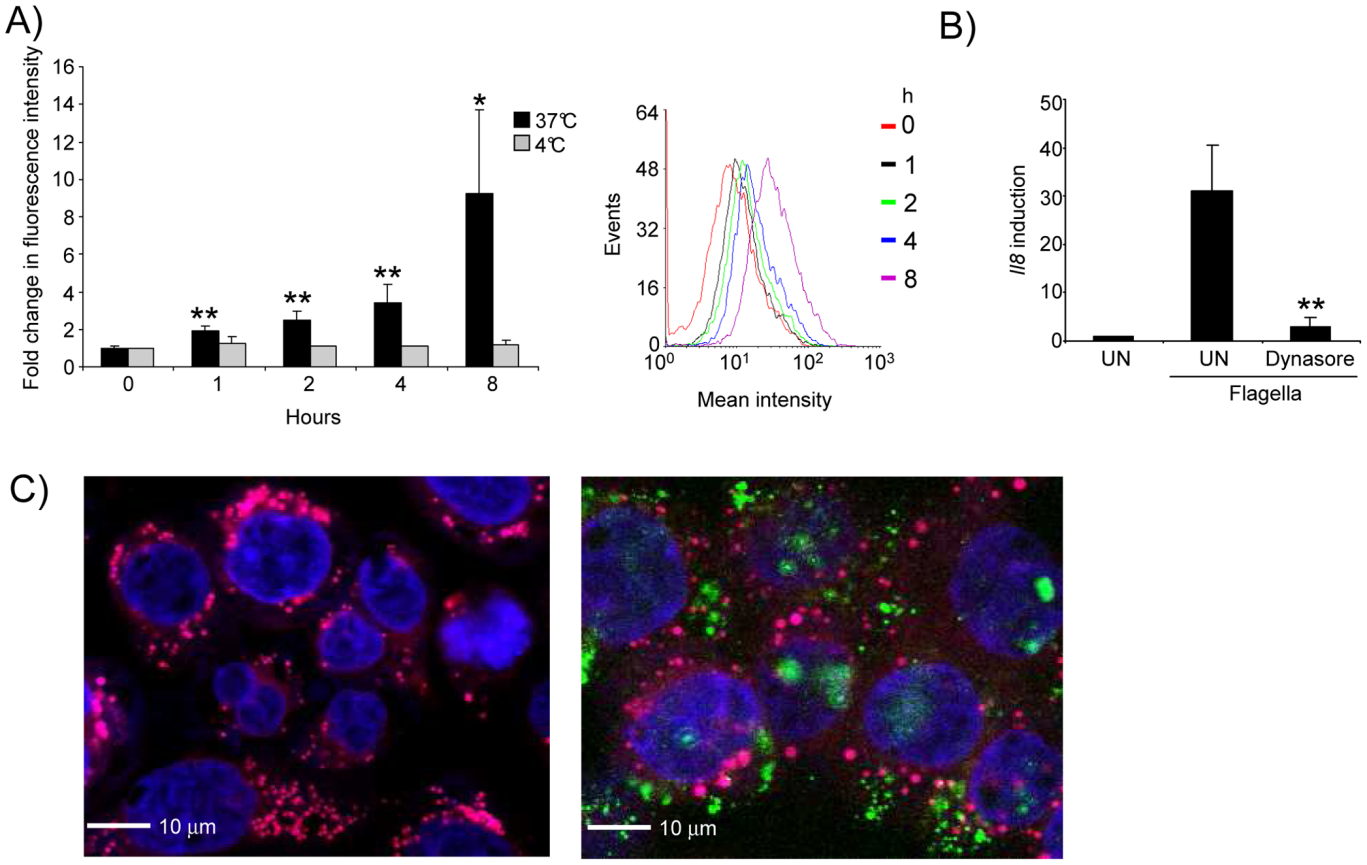
Flagella are internalized by epithelial cells. A) 16HBE cells were incubated with fluorescently labeled flagella for the indicated times and temperatures and analyzed by flow cytometry. A histogram representation of the data showing the increase in fluorescence intensity over time is also shown. B) 16HBE cells were incubated with flagella for 4 h in the presence or absence of dynasore. *Il8* levels were quantitated by qRT-PCR. C) Confocal microscopy images of 16HBE cells incubated with fluorescently labeled flagella (green) and stained with a nucleic acid dye (TOPRO3, blue) and lysosome dye (Lyso-ID, pink). *P<0.05, **P<0.01, students t test compared to untreated. Data are representative of two independent experiments. UN-unstimulated.

As flagella are often shed from growing bacteria, we examined whether airway cells endocytose flagella directly. We incubated Alex Fluor 488-labeled flagella with 16HBE airway epithelial cells and monitored fluorescence over time (Fig. 3A). There was significant uptake of labeled flagella at 37 degrees C (9.7-fold after 8 h, P<0.05) but not at 4 degrees C and this uptake increased over an 8 hour time period. Flagella uptake induced epithelial expression of *Il8* (Fig. 3B), which was completely abrogated in cells pretreated with dynasore (91% reduction, P<0.01), which blocks dynamin dependent GTPase activity associated with endocytosis [34]. Confocal images of the 16HBE cells clearly indicated the presence of flagella in the cytosol (Fig. 3C) and over the 4 hour incubation period (or 8 h, data not shown), little if any, co-localization of flagella and the lysosome was detected. Thus, flagella are rapidly endocytosed by airway epithelial cells in a dynamin-dependent fashion and persist within the cytosol.

### Endocytosed Flagella Activate TLR5-NF-κB Signaling in Airway Epithelial Cells

**Figure 4:**
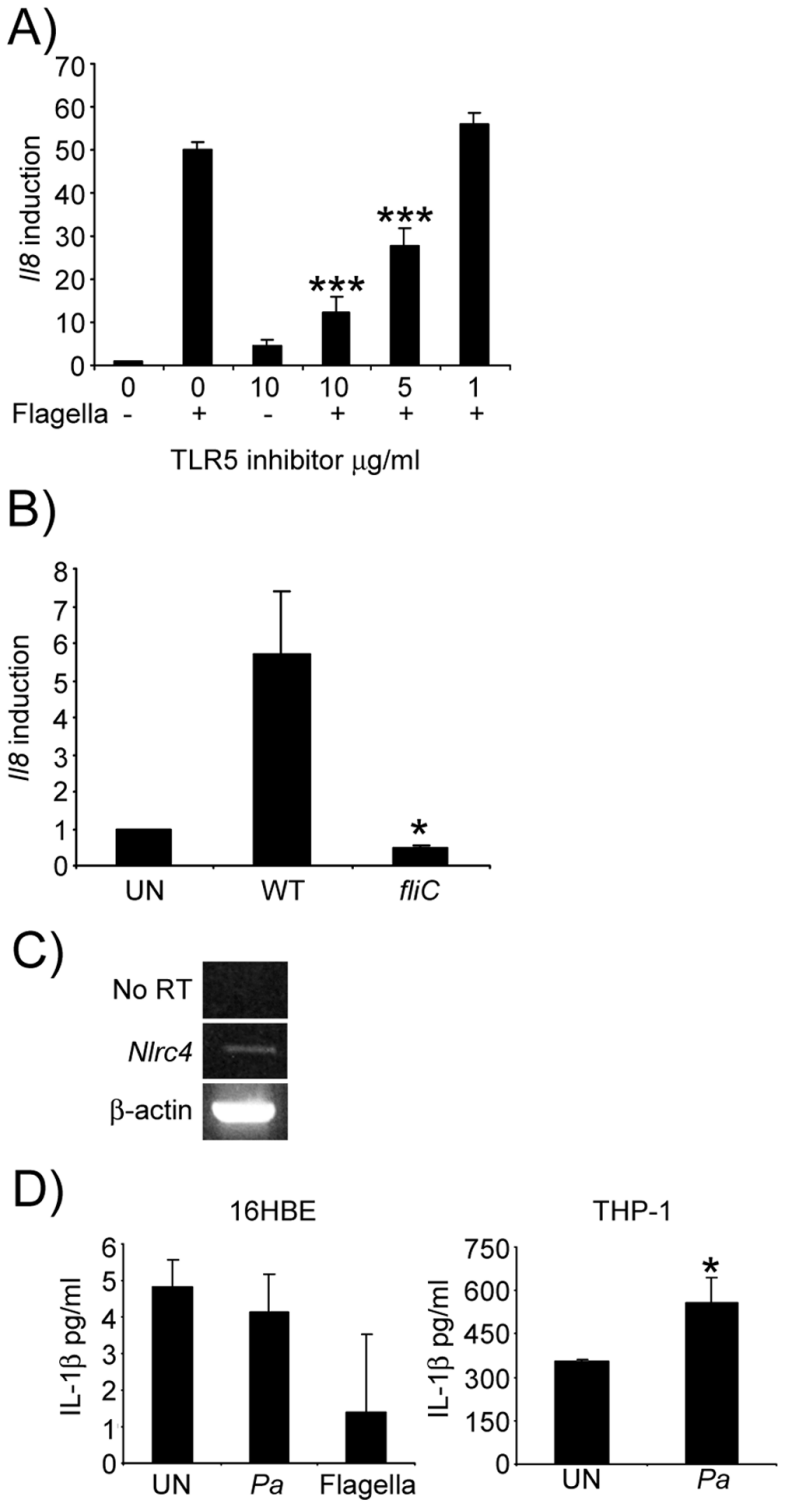
Signaling by flagella is TLR5 dependent. A) Flagella were incubated in the presence of varying concentration of a TLR5 inhibitory peptide and levels of *Il8* induction measured by qRT-PCR. ***P<0.001 compared to flagella without inhibitor. B) 16HBE cells were incubated with WT and *fliC* null strains of *P. aeruginosa* for 4 h before RNA was extracted and *Il8* levels quantitiated by qRT-PCR. C) RT-PCR of the *NlrC4* gene from 16HBE cells. D) Production of IL-1b by 16HBE and THP-1 cells in response to purified flagella and *P. aeruginosa (Pa*). *P<0.05, compared to WT stimulated cells. N = 3, Data are representatives of two independent experiments.

Airway epithelial cells induce *Il8* in response to purified *P. aeruginosa* flagella [16]. To document the involvement of TLR5 in this response we preincubated 16HBE cells with a TLR5 antagonist peptide before stimulation with flagella. In the absence of the TLR5 inhibitor flagella stimulated a 50-fold induction in *Il8* transcription that was decreased in a dose-dependent fashion by blocking TLR5 (Fig. 4A). To confirm the importance of flagella in stimulating epithelial signaling, we demonstrated 92% less (P<0.05) epithelial *Il8* induction by the *fliC* null mutant as compared with WT *P. aeruginosa* PAK. The involvement of epithelial inflammasome signaling was also investigated. The presence of an *Nlrc4* transcript was readily detected in the 16HBE cells, however IL-1ß expression was not detected following 2–24 hours of stimulation with flagella or *P. aeruginosa* (Fig. 4D). This is in contrast to the observed production of IL-1 ß in THP-1 cells in response to *P. aeruginosa* (Fig. 4D).

**Figure 5:**
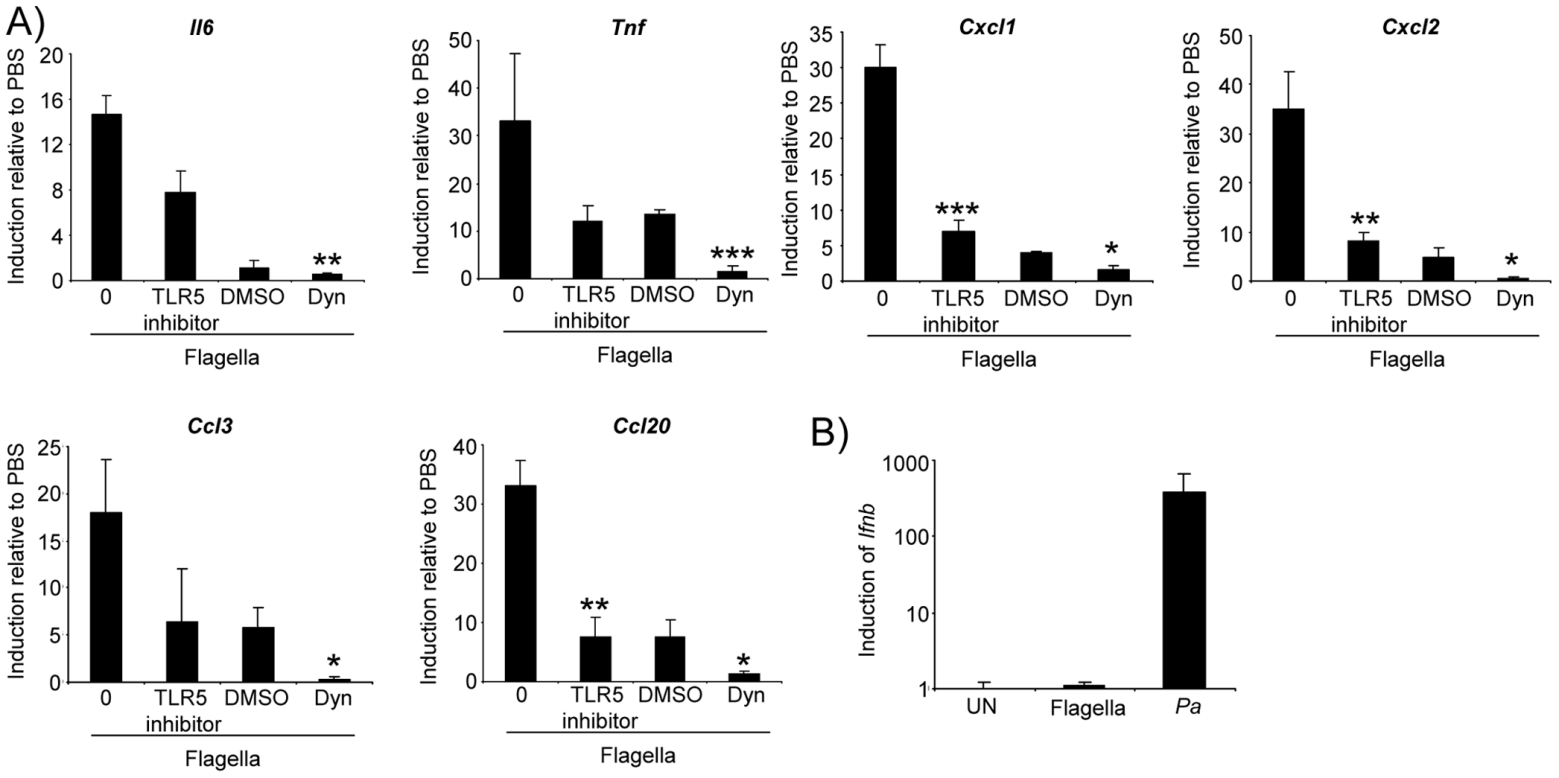
*P. aeruginosa* flagella activate TLR5-NF-κB signaling in airway epithelial cells. A) Purified *P. aeruginosa* flagellin was applied to 16HBE cells for 4 h before RNA was extracted and gene levels quantitated by qRT-PCR. *P<0.05, **P<0.01, ***P<0.001, TLR5 inhibitor is compared to flagella alone while dynasore is compared to the DMSO alone control (students t test). N = 3 and is representative of two independent experiments. Dyn-dynasore. B) Primary murine nasal epithelial cells were stimulated with purified flagella or *P. aeruginosa* (*Pa*) for 4 h before levels of *Ifnb* were quantitated by qRT-PCR.

To further establish the consequences of flagellin activation of epithelial TLR5 we monitored the expression of 84 cytokine and chemokine genes on 16HBE cells pre-treated with buffer, the TLR5 inhibitor, or dynasore (Fig. 5A). There was over 30-fold induction of epithelial TNF expression, which was decreased by 64% in the presence of the TLR5 inhibitor and completely blocked by dynasore (P<0.001), as was the induction of IL-6. The neutrophil chemoattractants, CXCL1 and CXCL2 were each induced by over 30-fold as was the macrophage chemoattractant CCL20 (Fig. 5A). The neutrophil chemokine CCL3 was also induced by 18-fold (Fig. 5A). All of the cytokine gene expression observed to be induced by flagella was inhibited in the presence of the TLR5 inhibitor as well as by dynasore (Fig. 5A). We did not observe any TRIF-dependent gene expression (such as *Ifnb*), which is readily produced in murine nasal epithelial cells (Fig. 5B). Thus, epithelial endocytosis of flagella initiates the major proinflammatory stimulus of epithelial cells via production of TLR5-NF-κB-dependent macrophage and neutrophil chemokines.

## Discussion

Bacterial flagella are sensed by many different types of cells in the airway and can interact with a number of discrete receptors. In these studies we observed that airway epithelial cells have a major signaling role in the initial response to flagellin. The predominant response of the airway epithelial cells to *P. aeruginosa* flagella, was endocytosis, activation of TLR5 signaling and expression of neutrophil and macrophage chemokines. These observations agree with recent studies demonstrating the induction of IL-8 and subsequently Muc5AC and host defense genes in airway cells in response to flagella [35], [36]. Based on published studies indicating that processed flagellins actually contain the relevant TLR5-epitope, we expect that endocytosed flagella are broken down in the cytoplasm to flagellin by available proteases, to expose the immunostimulatory domain [19], [20], [37], [38]. This contrasts with the gut where TLR5 is located basolaterally and therefore only senses organisms that have invaded through the epithelial layer [18]. Flagellin can also be internalized by intestinal epithelial cells however, the signaling consequences of this outside of IL-8 production are unknown [39]. Thus the epithelium is able to sense shed flagella from bacteria accumulating in the airway lumen, even in the absence of bacterial invasion or disruption of the epithelial barrier. This is especially relevant for patients with airway colonization with *P. aeruginosa*, either acutely as in VAP or in cystic fibrosis patients that are chronically colonized with *P. aeruginosa* and are known to have aberrant neutrophils [40], [41].

Bacterial components can induce changes in the airway epithelium to facilitate both bacterial and host cell migration. Intact *P. aeruginosa* through the expression of pilin adhesins and TTST are able to cleave tight junction proteins and invade through the paracellular spaces to reach the basolateral aspects of the polarized airway epithelium [31], where in vitro studies suggest that attachment occurs [5]. PAMPs of *P. aeruginosa* such as lipoteichoic acid and the cell wall lipoproteins sensed by TLR2 induce cleavage of tight junction proteins, occludin and ZO1, that facilitate neutrophil transmigration to the site of infection in the airway [33]. However, in contrast to lipoproteins, we did not observe that flagella, by themselves, were able to change the integrity of the epithelial barrier. The presence of a functional flagellum was required for epithelial transmigration, highlighting the cooperative effort of multiple *P. aeruginosa* virulence factors (type III toxins and flagella) in invasion. Likewise, the host signaling induced as a result of flagellin stimulation, proinflammatory cytokines and leukocyte chemokines, demonstrates the coordinated action of the innate immune system in response to pathogens. Flagellin along with other PAMPS induce leukocyte chemokines while TLR2 can facilitate the junctional changes necessary for leukocyte transmigration.

In addition to the contribution of flagella for *P. aeruginosa* transepithelial invasion, isolated flagella were found to be the major stimuli for epithelial proinflammatory chemokine and cytokine expression. Given the ability of rhamnolipid to induce shedding of flagellin from *P. aeruginosa* [19], [20], [37], [38], there is apparently substantial epithelial exposure to flagella during chronic airway infection. Unexpectedly we observed that internalization of isolated flagella is highly efficient and provides a potent stimulus for epithelial gene expression. This suggests that the airway epithelium serves a major role in surveillance of bacterial PAMPs and rapidly recruits phagocytes in response to detection and endocytosis of flagella. This endocytosis of flagella by epithelial cells results in chemokine expression, but does not result in epithelial inflammasome activation, in contrast to the requirement for TLR5 in internalization and IL-1β production in macrophages [42]. Exactly how flagella are processed in the epithelium remains unclear.

Our observations that epithelial signaling via TLR5 initiates neutrophil and macrophage chemokine production are consistent with in vivo studies of *P. aeruginosa* infection in *Tlr5* ^−/−^ mice [21]. *Tlr5^−/−^* mice were not significantly impaired at 24 hours post *P. aeruginosa* inoculation, but they did exhibit reduced initial inflammatory inflammation consistent with our signaling data. Similarly, mouse chimera studies have also demonstrated that neutrophil recruitment in response to flagellin is highly dependent upon radioresistant cells [43]. The importance of epithelial TLR5 has also been observed in allergic models of asthma using flagellin as a primer for allergic responses [44]. Although the flagellin-TLR5 interaction has not been shown to be essential in the resolution of *P. aeruginosa* pneumonia [21], epithelial recognition of flagellin contributes to pathogen clearance, and does not induce the more proinflammatory and damaging signaling associated with activation of the NLRC4 inflammasome [12], [13], [14], [15]. Given the redundancies of proinflammatory signaling mechanisms in the airway and the multiple PAMPs released by *P. aeruginosa*, epithelial recognition of flagellin appears to assist in the early recognition of potential pathogens and the initial recruitment of professional phagocytes to the airway.

## Materials and Methods

### Cell Lines, Bacterial Strains and Reagents

16HBE human airway epithelial cells (D. Gruenert, California Pacific Medical Center Research Institute [31], [45], [46]) were grown in MEM medium with 10% heat inactivated fetal bovine serum, 2 mM L-glutamine, penicillin and streptomycin. *P. aeruginosa* PAK and its derivatives were grown in LB broth. Green fluorescent protein (GFP) expressing strains of *P. aeruginosa* were grown in LB containing 300 µg/ml of carbenicillin. Mouse monoclonal antibodies to E-cadherin were used (BD Biosciences) along with Alexa Fluor conjugated secondary antibodies (Life Technologies). The TLR5 inhibitory peptide (H-18, Santa Cruz) was used at the indicated concentrations, typically 10 µg/ml unless otherwise stated. Dynasore [34] was suspended in DMSO and incubated an hour prior to stimulation at 80 µM. Primary murine nasal epithelial cells isolated from nasal septa were cultured as described elsewhere [47], [48]. THP-1 cells were grown in RPMI 1640 medium with antibiotics. The day before stimulation cells were exposed to 10 nM of PMA.

### Flagella Preparation

Flagella were purified as described previously [16] with the modification of growing cultures for 24 h in M9 minimal medium. After purification preparations were run through an endotoxin removal column (Thermo Scientific). Fluorescent labeling of flagella was conducted using the Alex Fluro 488 protein labeling kit (Life Technologies). Cells were typically stimulated with 10 µg/ml of flagella unless otherwise stated.

### Epithelial Permeability and Bacterial Transmigration

Bacterial transmigration assays were performed as described previously [31]. Briefly polarized 16HBE epithelial cells were stimulated for 4 h with 5×10^7^ cfu/ml of *P. aeruginosa* from an overnight grown culture in LB broth. 3, 000 MW dextran was added 1 h and fluorescence in the basal compartment measured at 485 nm excitation and 535 nm emission on a SpectroFluor Plus fluorimeter (Tecan). Bacteria present in the basal compartment were enumeration by serial dilution and plating on LB agar. Transepithelial resistance across the polarized monolayer was measured using a Millicell-ERS (Millipore).

### Confocal Microscopy

16HBE were stained as described previously [31] with cells permeabilized with 0.2% Triton X-100. Staining was performed at room temperature using E-cadherin antibodies (BD Biosciences) diluted 1∶100 followed by Alex Fluor 647 secondary antibody (Life Technologies) 1∶1000. Filters were removed from Transwells and mounted onto glass slides using Vectashield (Vector Laboratories). Imaging with purified flagella was performed using glass chamber slides (Millicell EZ slide, Millipore). TOPRO3 (Life Technologies) and Lyso-ID (Enzo) were applied 30 min before the end of the flagella stimulation. Cells were washed and fixed with 4% paraformaldehyde.

### RNA Analysis

Cells were typically stimulated with 10 µg/ml of purified flagella for 4 h unless otherwise stated. After stimulation cells were lysed using RNA lysis buffer and RNA extracted as per instructions of the PureLink RNA mini kit (Life Technologies). DNA was removed using DNAfree (Life Technologies) before cDNA synthesis using the High Capacity cDNA Reverse Transcription Kit (Applied Biosystems). qRT-PCR was performed using Power SYBR Green (Applied Biosystems) as described previously [47]. Stimulation of 16HBE cells with *P. aeruginosa* for RNA analysis was conducted for 4 h at an MOI of 20. qRT-PCR arrays (Human cytokines and chemokines, SABiosciences-Qiagen) were performed according to the manufacturers instructions but using cDNA and SYBR Green as described above. Sequences for oligonucleotide primers are as follows: β-actin, sense 5′ TCCTCCCTGGAGAAGAGCTAC 3′, antisense 5′ TAAAGCCATGCCAATCTCATC 3′; *Il8*, sense 5′ GTGCAGTTTTGCCAAGGAGT 3;, antisense 5′ CTCTGCACCCAGTTTTCCTT 3′; *Il6*, sense 5′ AAGAGTAACATGTGTGAAAGC 3′, antisense 5′ CTACTCTCAAATCTGTTCTGG 3′; *Tnf*, sense 5′ GAGTGACAAGCCTGTAGCCCA 3′, antisense 5′ GAATGATAAAGTAGACCTGCC 3′; *Cxcl1*, sense 5′ AGTCATAGCCACACTCAAGAATGG 3′, antisense 5′ GATGCAGGATTGAGGCAAGC 3′; *Cxcl2*, sense 5′ CTGCGCCCAAACCGAAGTCATA 3′, antsense 5′ TTCAGGAACAGCCACCAATAAGC 3′; *Ccl3*, sense 5′ TGCTGCTTCAGCTACACCTC 3′, antisense 5′ TTTCTGGACCCACTCCTCAC 3′; *Ccl20*, sense 5′ CTGGCCAATGAAGGCTGTGA 3′, antisense, 5′ GAAACCTCCAACCCCAGCAA 3′ and *NlrC4*, sense 5′ GCAAAGGCAAGTCCACTCTG 3′, antisense, 5′ CCATGAATGTCTGCTTCCTG 3′. Murine β-actin and *Ifnb* primers have been described elsewhere [49].

### FACS Analysis

Fluorescently labeled flagella was incubated with 16HBE airway epithelial cells for various times, dissociated from plates using 2% EGTA in PBS and suspending cells in 1% paraformaldeyde. Cells were analyzed on a FACScalibur (Becton Dickinson) using CellQuest software (version 3.3; BD). Cells were gated on their side and forward scatter and FITC levels were quantitated.

### Ethics Statement

All mouse experiments were performed under the guidelines of the Institutional Animal Care and Use Committee of Columbia University.
